# A Rare Form of Cardiac Disease

**Published:** 1870-11

**Authors:** N. S. Davis

**Affiliations:** Chicago


ARTICLE XXXVI.

### A RARE FORM OF CARDIAC DISEASE.

BY N. S. DAVIS, M.D., Etc., Etc., Chicago.

[Presented to the Chicago Medical Society.]

Mr. S., aged about 26 years; American; married; applied to me for advice six or eight months since. He was spare in flesh, anemic in appearance, yellow or jaundiced in the color of the skin and conjunctiva; and complained of indigestion; general muscular weakness; shortness of breath on attempting to exercise; irregular pains in the right hypochondriac region; and occasional sharp pains in the region of the heart. His bowels were irregular, sometimes loose and at others costive; urine scanty, dark brown, and often mixed with blood. Physical exploration revealed no evidence of disease of the lungs, or enlargement of any of the abdominal viscera. The rythm of the heart was normal, but the first sound was accompanied by a peculiar *flap*, a quality not easily described. It differed from the bellows sounds that accompany either extreme æenemia, or valvular roughness. The systole was short and quick, with a occasional intermission. He had completed a Collegiate and Theological course of study, supporting himself by teaching music. While making some physical exertion in the open air, at the age of 12 years, he suddenly fell as though seized with spasms. But the general failure of his health was not much manifest until he was in the midst of his studies in College and teaching at the same time; and hence the failure had been attributed to over-tasking himself, both mentally and physically. By proper regulation of diet and exercise, aided by mild tonics, the functions of the stomach and kidneys were much improved, and he continued to attend to some business until about the first of August, 1870. He was then attacked with symptoms of dysentery, coupled with a few paroxysms of intermittent fever, which appeared to greatly prostrate him, and all efforts at exercise were attended with palpitation, shortness of breath, sense of exhaustion, etc., with momentary severe pains in his chest.

A few days treatment relieved the fever paroxysms, and improved the state of the bowels, but he continued very week, and the action of the heart quite irregular, with some œdema and coldness of the extremities. While in this condition he was attacked with copious hemorrhage from the bowels, which proved speedily fatal.

Twenty-four hours after death a *post mortem* examination was made by Prof. H. W. Boyd, assisted by Dr. Ward and a Student. They reported no morbid appearance in the viscera of the abdomen except congestion, and perhaps slight softening of portions of the mucous membrane of the ilum and colon, and increased vascularity, with some fatty degeneration of the kidneys. The liver and spleen were reported healthy and of normal size. The lungs appeared healthy except numerous deposits of dark pigmenty matter in the upper and anterior parts. The heart was about the natural size, pale in color, with a little more accumulation of fat over its base than usual. On cutting through the walls of the ventricles the muscular structure was found thinner and paler than natural on both sides, but more especially was this the case in the walls of the right ventricle, where the muscular structure had been partially replaced by fatty tissue. A white tough fibrous clot or mass was found in the left ventricle, which continued into the aorta four or five inches, but did not appear to be attached or adherent to any part of the cardiac structure. In the right ventricle was found another mass, more white and compact than the one in the left. It was nearly cylindrical in form, and extended from the apex of the ventricle into the pulmonary artery, beyond the point where the latter vessel was severed in removing the heart from its position in the chest. This body was closely attached to and completely identified with the free edge of the anterior lobe of the tricuspid valve, but was free from all other attachments. The semi-lunar valves of both sides, the mitral, and the lobes of the tricuspid appear entirely free from thickening, or signs of disease, except just the edge to which the contained body is attached. The heart was presented and examined by the members present with much interest. Was the body in the right ventricle and pulmonary artery a true excresence or polypoid growth from the edge of the tricuspid valve? And was it this that had been for many years embarrassing the circulation and respiration, and rendering a satisfactory diagnosis difficult? Or was this a simple fibrinous formation, (a thrombus), and the apparent identification with the edge of the tricuspid valve a mere accidental adhesion. The latter is probably the true state of the case. Yet, the firmness of the adhesions; the cylindrical form, and density of the thrombus, together with the preceding history of the patient, indicate that it had been in existence a long period of time. That in the left cavity is evidently of more recent formation.

